# Synergistic Effect of Nanofluids and Surfactants on Heavy Oil Recovery and Oil-Wet Calcite Wettability

**DOI:** 10.3390/nano11071849

**Published:** 2021-07-17

**Authors:** Jinjian Hou, Lingyu Sun

**Affiliations:** 1 School of Chemical Engineering and Technology, Tianjin University, Tianjin 300072, China; sunlingyu4321@163.com; 2 National Engineering Research Centre for Distillation Technology, Tianjin 300072, China; 3 Collaborative Innovation Center of Chemical Science and Engineering, Tianjin 300072, China

**Keywords:** nanofluids, surfactants, enhanced oil recovery, wettability

## Abstract

In recent years, unconventional oils have shown a huge potential for exploitation. Abundant reserves of carbonate asphalt rocks with a high oil content have been found; however, heavy oil and carbonate minerals have a high interaction force, which makes oil-solid separation difficult when using traditional methods. Although previous studies have used nanofluids or surfactant alone to enhance oil recovery, the minerals were sandstones. For carbonate asphalt rocks, there is little research on the synergistic effect of nanofluids and surfactants on heavy oil recovery by hot-water-based extraction. In this study, we used nanofluids and surfactants to enhance oil recovery from carbonate asphalt rocks synergistically based on the HWBE process. In order to explore the synergistic mechanism, the alterations of wettability due to the use of nanofluids and surfactants were studied. Nanofluids alone could render the oil-wet calcite surface hydrophilic, and the resulting increase in hydrophilicity of calcite surfaces treated with different nanofluids followed the order of SiO_2_ > MgO > TiO_2_ > ZrO_2_ > γ-Al_2_O_3_. The concentration, salinity, and temperature of nanofluids influenced the oil-wet calcite wettability, and for SiO_2_ nanofluids, the optimal nanofluid concentration was 0.2 wt%; the optimal salinity was 3 wt%; and the contact angle decreased as the temperature increased. Furthermore, the use of surfactants alone made the oil-wet calcite surface more hydrophilic, according to the following order: sophorolipid (45.9°) > CTAB (49°) > rhamnolipid (53.4°) > TX-100 (58.4°) > SDS (67.5°). The elemental analysis along with AFM and SEM characterization showed that nanoparticles were adsorbed onto the mineral surface, resulting in greater hydrophilicity of the oil-wet calcite surface, and the roughness was related to the wettability. Surfactant molecules could aid in the release of heavy oil from the calcite surface, which exposes the uncovered calcite surface to its surroundings; additionally, some surfactants adsorbed onto the oil-wet calcite surface, and the combined role made the oil-wet calcite surface hydrophilic. In conclusion, the study showed that hybrid nanofluids showed a better effect on wettability alteration, and the use of nanofluids and surfactants together resulted in synergistic alteration of oil-wet calcite surface wettability.

## 1. Introduction

Carbonate asphalt rocks contain heavy oils, and many methods have been used to enhance heavy oil recovery from these rocks [1,2,3,4], such as hot-water-based extraction (HWBE) [5], solvent extraction [6,7,8], and pyrolysis, among others. However, these traditional methods have many disadvantages [9], including their high energy consumption and associated environmental pollution.

Recently, the unique effect of nanofluids in enhancing oil recovery has been revealed [10,11]. Cheraghian Goshtasp et al. [12] showed that nanoparticles could effectively alter oil/water interfacial tension alteration and enhance oil recovery. TiO_2_ nanoparticles could effectively improve surfactant-enhanced oil recovery [13]. Furthermore, the use of surfactants in heavy oil recovery has also shown advantages [14,15]. However, there are few studies on the possible synergistic effect of using both nanofluids and surfactants in heavy oil recovery, and the mechanism is unclear.

Wettability is the measurement of a liquid’s ability to interact with other fluids and/or solid surfaces. The wettability of a mineral surface is usually evaluated in terms of contact angle by dripping water onto a mineral surface. If the contact angle is lower than 90°, the mineral’s surface is considered hydrophilic. However, if the contact angle is higher than 90°, the mineral’s surface is hydrophobic. Of the two, hydrophilic surfaces are beneficial for heavy oil recovery.

Nowadays, nanofluids are widely used to enhance the oil recovery process [11,16,17]. Much research has indicated that the reasons for their enhancement of oil recovery are related to the wettability alteration of minerals. Cheraghian et al. reviewed the nanotechnology of enhanced oil recovery processes, and wettability was considered the key mechanism determining the enhancement of this process [17]. Hou et al. [18] assessed the effect of silica nanoparticles on the alteration of the wettability of oil-wet carbonate surfaces, studying the effect of the monovalent metal ion. The results showed that the silica nanoparticles could effectively adsorb onto the calcite surface, which made the surface water-wet, and that Na^+^ played an important role during the adsorption process. Sarmad Al-Anssari et al. [19] studied the effect of silica nanofluids on wettability under reservoir conditions, and the results indicated that silica nanofluids could effectively alter oil-wet calcite wettability under conditions of high temperature, pressure, and salinity, and that the nanoparticle adsorption phenomenon was mainly irreversible. Ali Esfandyari Bayat et al. [20] studied the effect of different oxide nanoparticles in enhancing oil recovery from limestone at several temperatures, and the results showed that the contact angles of the mineral surface treated by Al_2_O_3_, TiO_2_, and SiO_2_ nanofluids were 71° ± 2°, 57° ± 2°, and 26° ± 2°, respectively. The research of Iman Nowrouzi et al. [21] showed that a higher concentration and smaller particle sizes of TiO_2_ decreased the contact angle and thus improved heavy oil recovery.

Although many papers have focused on the effect of nanofluids on mineral surface wettability, the conclusions are contradictory, at least when comparing different conditions. That is, most research has only focused on individual factors, but nanofluids, nanoparticle species, temperature, salinity, concentration, hybrid nanoparticles, and other parameters should be studied under the same conditions. Additionally, there is little research on the synergistic effect of nanofluids and surfactants on wettability alteration. Furthermore, there is no research on the effect of nanofluids and surfactants in enhanced oil recovery by the hot-water-based method (HWBE) because this process has mostly been used for sandstone asphalt rocks. Last but not least, details regarding the effect of nanofluids on mineral surface wettability are unclear [22,23,24], as in most reports, wettability has only been studied through contact angle, and elemental and morphological analyses are lacking, which are required for establishing a detailed mechanism.

The purpose of this research was to (i) study the synergistic effect of nanofluids and surfactants on heavy oil recovery from carbonate asphalt rocks using the HWBE process; (ii) study the effect of aging time, nanofluid species, concentration, temperature, salinity, and hybrid nanofluids on oil-wet calcite wettability and perform an elemental analysis and a morphological analysis; and (iii) study the synergistic effect of nanofluids and surfactants on oil-wet calcite wettability.

## 2. Materials and Methods

### 2.1. Materials

Chemical reagents of analytical grade, including toluene, hydrochloric acid, and sodium hydroxide, were purchased from Tianjin Jiangtian Technology Co (Tianjin, China). Ltd. Heavy oil was extracted from carbonate asphalt rocks. The carbonate asphalt rocks were obtained from Buton Island, Indonesia. SiO_2_, γ-Al_2_O_3_, TiO_2_, MgO, and ZrO_2_ nanoparticles (analytical-grade purity) were from Aladdin Reagent Co., Ltd., Shanghai, China. CTAB, SDS, TX-100, sophorolipid, and rhamnolipid were purchased from Aladdin Reagent Co., Ltd., China. Water was deionized in the laboratory for further use.

### 2.2. Heavy-Oil–Solid Separation Process

Nanofluids and biosurfactants were used to extract bitumen from oil sands. Different kinds of aqueous solutions (deionized water, nanofluid, surfactant, bionanofluid) were prepared. The detailed operational steps were as follows: First, the oil sands (50 g) were crushed, sieved, and weighed. The pH of the aqueous solutions was adjusted to 8.50. Second, 50 g of oil sand ores were transferred into a Denver flotation cell. Third, 900 mL of aqueous solution (52 °C) were poured into the Denver flotation cell. The condition process was under 45 °C for 5 min, while 150 mL/min air was injected into the flotation cell. Finally, after a flotation time of 15 min, the froth was collected for analysis using the Dean–Stark apparatus. The bitumen recovery was then calculated.

### 2.3. Wettability Alteration Experiment

**Figure 1:**
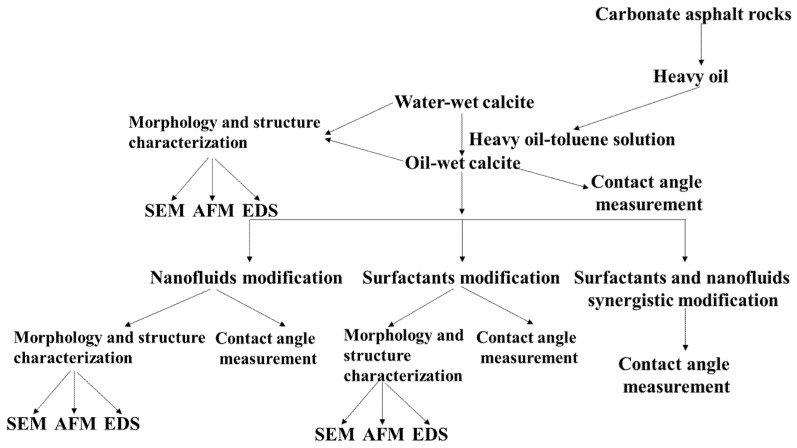
Scheme of the experiment procedure.

The detailed experiment procedure is shown in Figure 1. Firstly, heavy oil was extracted from carbonate asphalt rocks, and a heavy-oil–toluene solution was configured. Then, water-wet calcite was soaked in the heavy-oil–toluene solution, which rendered the oil-wet calcite surface hydrophobic. The contact angle of the oil-wet calcite surface was measured, and the morphology and structure of the treated oil-wet calcite surface were characterized by SEM–EDS and AFM. The oil-wet calcite was then treated by either nanofluids, surfactants, or a nanofluid–surfactant hybrid solution. Finally, following these modifications, the water contact angle on oil-wet calcite was measured, and the morphology and structure assessed.

### 2.4. Water-Wet Calcite Modification

The carbonate asphalt rocks were ground and sieved, and 20 g samples were weighed out. Heavy oil was extracted from the carbonate asphalt rocks by Soxhlet extraction. The heavy oil was dissolved into toluene with the application of ultrasonication. Thus, a 10 wt% heavy-oil–toluene solution was formed.

Taking clean calcite surfaces as the samples, the flat debris was chosen as the experiment slice. Acetone, ethanol, and deionized water were used to clean the calcite surface, and a clean water-wet calcite surface was thus formed. The contact angle was measured and the water-wet calcite surface analyzed by SEM–EDS and AFM.

Briefly, the 10 wt% heavy-oil–toluene solution was added into a 50 mL screw top bottle, and water-wet calcite was soaked in the heavy oil. After 15 days, the oil-wet calcite was taken out and placed onto clean paper. After the toluene had volatilized, aging oil-wet calcite was left. The oil-wet calcite surface wettability was then measured, and the morphology and structure analyzed. The influence of different soaking times on the water-wet contact angle was assessed.

### 2.5. Nanofluid-Treated Oil-Wet Calcite

Briefly, 0.1 wt% SiO_2_, MgO, TiO_2_, γ-Al_2_O_3_, and ZrO_2_ nanofluids were prepared. The nanofluid concentrations and salinity were altered, and the nanofluids were distinguished. The aging oil-wet calcite was placed in the nanofluids and left for 3 days. The role of the various nanofluids on the calcite surface was assessed through analysis of the calcite morphology and structure.

### 2.6. Surfactant-Treated Oil-Wet Calcite

First, 500 ppm CTAB, SDS, TX-100, rhamnolipid, and sophorolipid solutions were prepared. Then, the aging oil-wet calcite was placed in the surfactant solutions and left for 3 days. The surfactants’ effect on the calcite surface was measured, and the calcite morphology and structure were analyzed.

### 2.7. Synergistic Treatment of Oil-Wet Calcite with Surfactants and Nanofluids

Briefly, 0.1 wt% TiO_2_, MgO, and SiO_2_ nanoparticles were dispersed into 500 ppm rhamnolipid, CTAB, and sophorolipid solutions to create surfactant–nanofluid mixtures. The aging oil-wet calcite was placed into the combined surfactant–nanofluid solutions and left to stand for 3 days. The surfactant–nanofluid solutions synergistically altered the oil-wet calcite surface wettability, and the oil-wet calcite surface morphology and structure were measured.

The contact angle effectively measures the wettability of minerals’ surface. For differently treated oil-wet calcite surfaces, the detailed experiment procedure was as follows: different aging oil-wet calcite samples were placed onto the sample stage, and the water drop was placed onto the samples and was fitted. Nanofluids treated the oil-wet calcite surface, as did surfactants, and the samples’ wettability was measured.

### 2.8. Morphology Analysis by SEM–EDS and AFM

For water-wet calcite surfaces and oil-wet calcite surfaces, SEM–EDS (S4800, Tokyo, Japan) was used to observe the differences in morphology and composition between them and to explore the surface elements’ differences. Furthermore, AFM measurement could effectively measure the calcite surface roughness. Thus, AFM was used to measure the effect of treatment with the different surfactants, nanofluids, and surfactant–nanofluid formulations on the surface roughness of the oil-wet calcite.

### 2.9. Zeta Potential Measurement

In order to explore the charge of different nanofluids, different surfactants, and the surfactant–nanofluids hybrid systems, the zeta potential (Malvern, mV, Shanghai, China) was measured.

## 3. Results

### 3.1. Nanoparticle Morphology Analysis

#### 3.1.1. SEM Analysis

**Figure 2:**
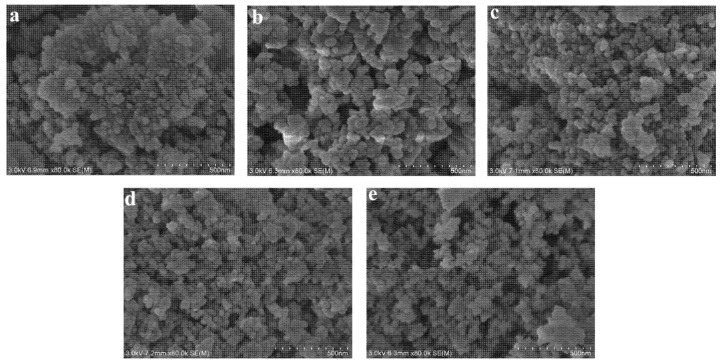
SEM images of (**a**) γ-Al_2_O_3_; (**b**) MgO; (**c**) TiO_2_; (**d**) ZrO_2_; (**e**) SiO_2_.

Figure 2 shows SEM images of the five types of nanoparticles studied. The five nanoparticles were generally round in shape, and the morphology among different nanoparticles was similar. The diameter of γ-Al_2_O_3_, MgO, TiO_2_, ZrO_2_, and SiO_2_ was 50 nm.

#### 3.1.2. TEM Analysis

**Figure 3:**
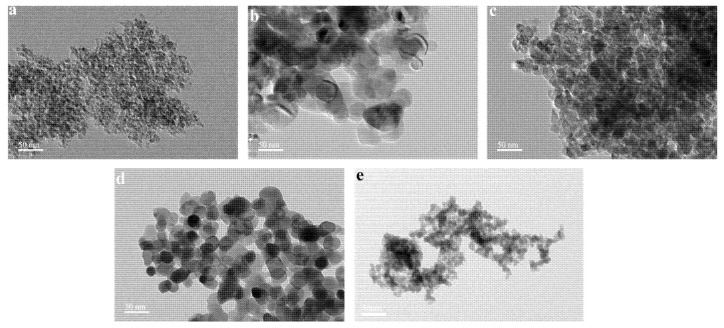
TEM images of (**a**) γ-Al_2_O_3_; (**b**) MgO; (**c**) TiO_2_; (**d**) ZrO_2_; (**e**) SiO_2_.

As shown in Figure 3, TEM analysis showed that the five nanoparticles generally appeared as irregular spheres. All nanoparticles showed weak dispersity and aggregated in aqueous solutions. The different nanoparticles showed different diameters which, in turn, result in different effects on oil recovery.

### 3.2. Synergistically Enhanced Oil–Solid Separation Using Surfactants and Nanofluids

**Figure 4:**
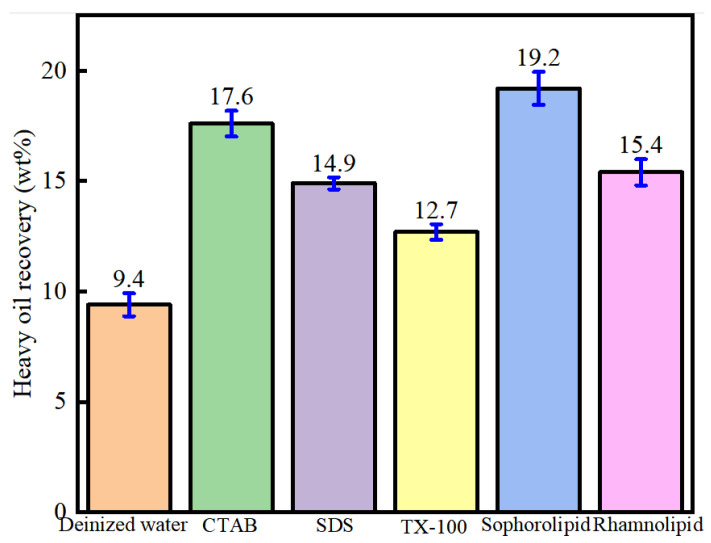
Heavy oil recovery using heavy-oil–carbonate-solid separation enhanced by five surfactants (500 ppm).

As shown in Figure 4, application of the five surfactants could effectively enhance heavy-oil–solid separation, and the oil recovery followed the order of sophorolipid (19.2 wt%) > CTAB (17.6 wt%) > rhamnolipid (15.4 wt%) > SDS (14.9 wt%) > TX-100 (12.7 wt%) > deionized water (9.4 wt%). Different surfactants had different effects on the enhancement of heavy-oil–solid separation, which was due to the fact that different surfactant molecules showed different effects on oil–water interface modification and oil–solid interface modification.

**Figure 5:**
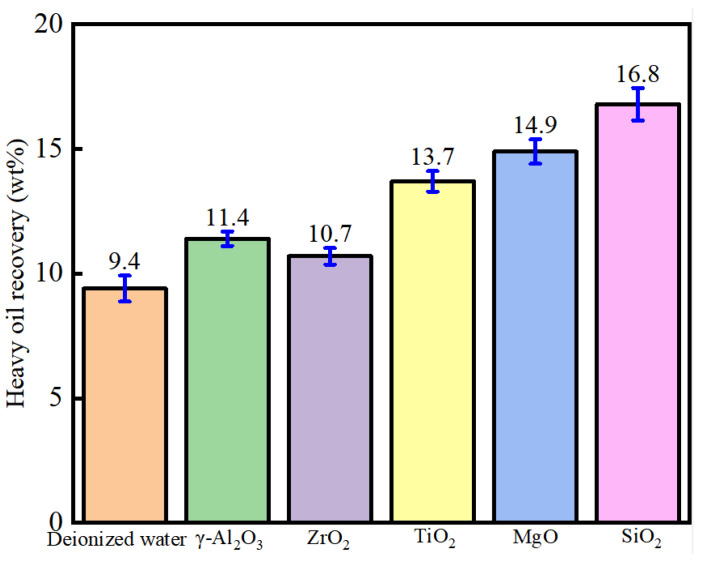
Heavy oil recovery using heavy-oil–carbonate-solid separation enhanced by five nanofluids (0.1 wt%).

Figure 5 shows that treatment with the 0.1 wt% γ-Al_2_O_3_, MgO, TiO_2_, ZrO_2_, and SiO_2_ nanoparticles showed an obvious effect in increasing heavy oil recovery. Compared to the deionized water without any nanoparticles, 0.1 wt% SiO_2_ nanofluid increased the heavy oil recovery from 9.4 to 16.8 wt%. MgO, TiO_2_, γ-Al_2_O_3_, and ZrO_2_ could increase heavy oil recovery to 14.9, 13.7, 11.4, and 10.7 wt%, respectively. The addition of nanofluids could effectively enhance heavy oil recovery, which was due to the fact that nanofluids could effectively alter the oil/water interface and the carbonate mineral surface. The different nanofluids had different effects on oil recovery, and different nanoparticles showed different characteristics on mineral surface adsorption, oil/water interface properties, and oil/solid interface modification.

**Figure 6:**
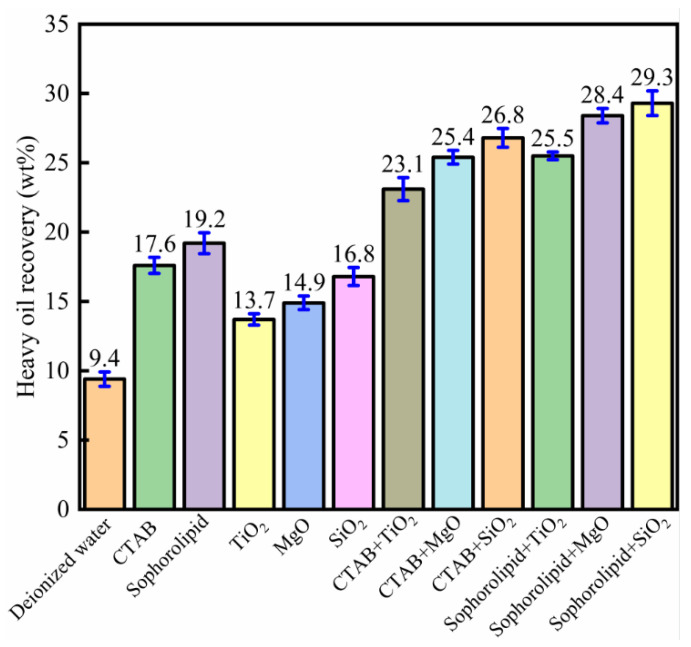
Heavy oil recovery from carbonate asphalt rocks using hot-water-based extraction synergistically enhanced with various nanofluid–surfactant solutions.

Figure 6 shows the effect of nanofluids–surfactants on oil recovery from carbonate asphalt rocks. Compared to the deionized water without nanoparticles and surfactants, when 0.1 wt% TiO_2_ was dispersed into 500 ppm CTAB, the oil recovery increased from 9.4 to 23.1 wt%, which was higher than the increase in oil recovery from treatment with CTAB alone (17.6 wt%) or TiO_2_ nanoparticles alone (13.7 wt%). The combinations of CTAB and TiO_2_, CTAB and MgO, CTAB and SiO_2_, sophorolipid and TiO_2_, sophorolipid and MgO, and sophorolipid and SiO_2_ showed obviously synergistic effects on heavy-oil–solid separation, whereby the oil recovery was higher than that when using nanofluid or surfactant alone. The use of SiO_2_ nanofluid with sophorolipid surfactant showed an optimal synergistic effect, in which the oil recovery increased to 29.3 wt%. The reasons for synergistic effects are as follows. On the one hand, the surfactant molecules could adsorb onto the nanoparticle surface, which increases the steric hindrance of nanoparticles and, thus, the stability of the nanofluid [25]. The increased nanofluid stability then results in increased oil recovery. On the other hand, surfactants and nanofluids could synergistically decrease oil/water interfacial tension, which helps improve oil recovery [12,26,27]. Some research has indicated that nanofluids and surfactants could synergistically increase structural disjoining pressure [28,29]. However, whether surfactants and nanofluids have a synergistic effect on wettability alteration was unknown and is the focus of this study.

Figure 6 shows that nanofluids and surfactants alone effectively enhance oil recovery from carbonate asphalt rocks, and surfactants and nanofluids have a synergistic effect on heavy oil recovery. As is known, wettability influences oil recovery. The more hydrophilic a mineral’s surface, the higher the oil recovery. In order to explore the mechanism underlying the synergistic effect of surfactants and nanofluids, their effect on wettability alteration was assessed through contact angle measurements. Calcium carbonate is the main component of carbonate asphalt rocks; therefore, calcite was chosen to represent the minerals of carbonate asphalt rocks [18,19,30]. Oil-wet calcite was prepared to represent the actual carbonate rock surface.

### 3.3. Water-Wet Calcite Surface Modification

#### 3.3.1. Aging Time on the Contact Angle

**Figure 7:**
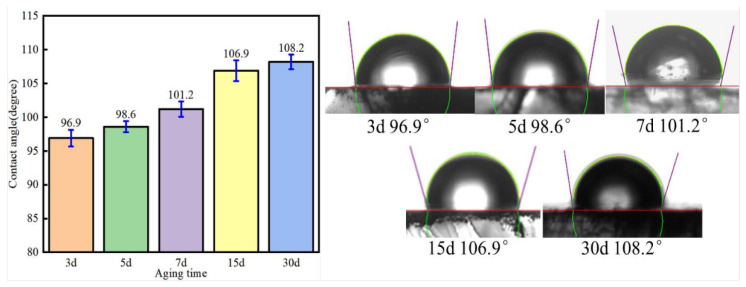
The aging time effect on the wettability of oil-wet calcite (25 °C).

**Figure 8:**
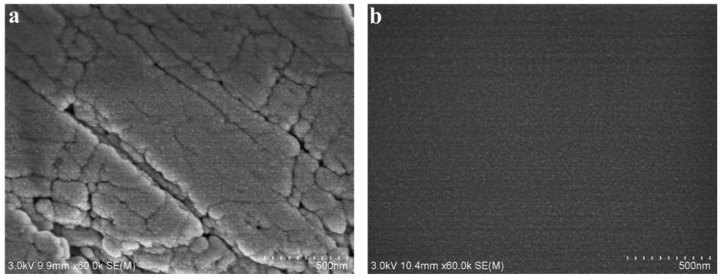
SEM images of (**a**) original water-wet calcite and (**b**) modified oil-wet calcite.

**Figure 9:**
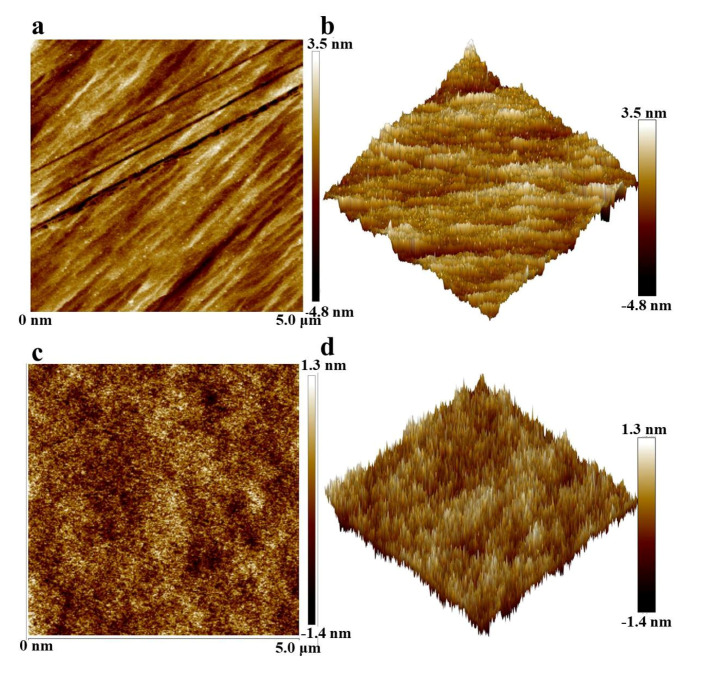
AFM images of (**a**,**b**) original water-wet calcite and (**c**,**d**) modified oil-wet calcite.

In order to render the water-wet calcite hydrophobic, the water-wet calcite was soaked in a heavy oil phase, and the heavy oil components adsorbed onto the water-wet calcite surface, making the surface more hydrophobic. The aging time effect on calcite wettability is shown in Figure 7. As the aging time increased, the contact angle on the mineral surface increased, which meant that the calcite hydrophobicity had increased. When the aging time was 3 days, the contact angle on the oil-wet calcite surface was 96.9°. When the aging time was increased to 15 days, the contact angle increased to 106.9°. However, when the aging time was increased further, the contact angle remained stable. The increase was small, and the contact angle was 108.2°, probably because the amount of heavy oil adsorbed remained stable, with the adsorption site being full after 15 days. When the heavy oil components adsorbed onto the calcite surface, the oil-wet calcite became flatter and smoother, as shown in Figure 8 and Figure 9. Here, the oil-wet surface showed a contact angle of 106.9°.

#### 3.3.2. Morphology Analysis

As shown in Figure 8, the original water-wet calcite surface was scaly, and the surface was rough. However, the modified oil-wet calcite surface was smooth, with low roughness. The main components of the water-wet calcite surface were CaCO_3_ and some other carbonate minerals. The water-wet calcite surface was uneven, and the CaCO_3_ was exposed to the surroundings. However, when the water-wet calcite was treated by heavy oil, the heavy oil molecules were adsorbed onto and covered the calcite surface. The oil-wet calcite surface roughness thus decreased. In the following experiment, the water contact angle on the oil-wet calcite was easily calculated.

As shown in Figure 9, the AFM results indicate that the water-wet calcite surface roughness was 4.8 nm, and the roughness of the oil-wet calcite surface was lower than that of the water-wet calcite surface, which was due to the fact that the oil components adsorbed onto the mineral surface, resulting in its significant decrease. Figure 9a shows that the original water-wet calcite surface was angular, which was due to the fact that the calcite had a hexagonal system, in which the lines are clear. Furthermore, Figure 9b shows the roughness of the water-wet calcite and the three-dimensional topography of the water-wet calcite surface; the water-wet calcite surface showed edges and corners. Figure 9c shows that the oil-wet calcite was smooth and flat; the oil components covered the calcite surface, which made the uneven surface flatter. Figure 9d shows the roughness of the oil-wet calcite surface, and the results show that the surface was flatter.

#### 3.3.3. Elemental Analysis

The water-wet calcite and oil-wet calcite SEM–EDS analysis results are shown in Table 1. The main elements of water-wet calcite are C (6.46 wt%), O (50.23 wt%), and Ca (43.31 wt%), and the C element mainly originated from CaCO_3_, at a low content of 6.46 wt%. The C element in oil-wet calcite reached an amount of 30.22 wt% and was from heavy oil. The elemental analysis indicated that heavy oil had adsorbed onto the water-wet calcite surface.

### 3.4. Nanofluids’ Effect on Oil-Wet Calcite Morphology and Structure

#### 3.4.1. Morphology Analysis

**Figure 10:**
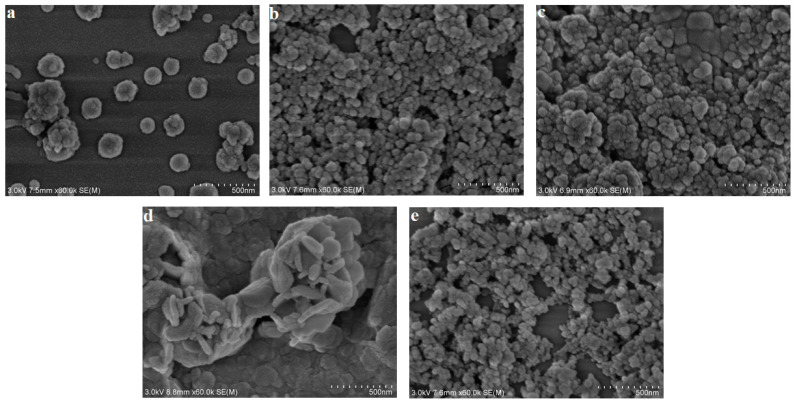
SEM images of adsorption effect of (**a**) γ-Al_2_O_3_, (**b**) ZrO_2_, (**c**) TiO_2_, (**d**) MgO, and (**e**) SiO_2_ on oil-wet calcite.

SEM images of the oil-wet calcites treated by five nanoparticles are shown in Figure 10. The SEM results indicate that the oil-wet calcite surfaces showed different degrees of uniformity and aggregation. The ZrO_2_, TiO_2_, and SiO_2_ nanoparticles were sphere-like, with a diameter of 50 nm. MgO nanoparticles showed aggregation into stripe-like shapes. The γ-Al_2_O_3_ nanoparticles were scattered, and the adsorbed amount was lower. Figure 10 shows that all nanoparticles could adsorb onto the oil-wet calcite surface. The nanoparticles’ adsorption effect, surface energy, and affinity influenced the oil-wet calcite surface wettability.

**Figure 11:**
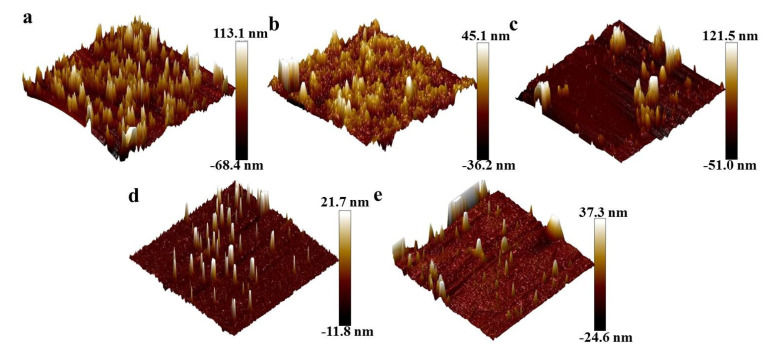
AFM images oil-wet calcite modified with (**a**) SiO_2_, (**b**) MgO, (**c**) ZrO_2_, (**d**) TiO_2_, and (**e**) γ-Al_2_O_3_.

Figure 11 shows the AFM images of oil-wet calcite surfaces treated with five different nanoparticles. The roughness values of the oil-wet calcite surfaces treated with SiO_2_, MgO, ZrO_2_, TiO_2_, and γ-Al_2_O_3_ were 113.1, 45.1, 121.5, 21.7, and 37.33 nm, respectively. The nanoparticle properties and the adsorption properties influenced the roughness of the oil-wet calcite surfaces. Higher roughness is beneficial to water-wet calcite [19,31]. In a previous study, Zhao indicated that the surface energy and roughness are related to mineral surface wettability [32].

#### 3.4.2. Elemental Analysis

The results from the EDS analysis of figures of oil-wet calcite modified using five nanofluids are shown in Table 2. For oil-wet calcite, the C, O, and Ca element content was 30.22, 27.49, and 42.29 wt%, respectively. The C and O elements were from heavy oil and CaCO_3_, and the Ca element was mainly from CaCO_3_. After nanofluid treatment of the oil-wet calcite, the sample surface’s C element content decreased, O element content increased, and the corresponding nanoparticles’ element occurred. When Al_2_O_3_ nanofluids were used to treat the oil-wet calcite surface, the sample’s surface showed the presence of C (7.56 wt%), O (50.34 wt%), Al (8.85 wt%), and Ca (33.25 wt%). The Al element content indicated that Al_2_O_3_ nanoparticles had adsorbed onto the oil-wet calcite surface. For other nanofluids, there were similar conclusions.

### 3.5. Surfactants’ Effect on Oil-Wet Calcite Morphology and Structure

#### 3.5.1. Morphology Analysis

**Figure 12:**
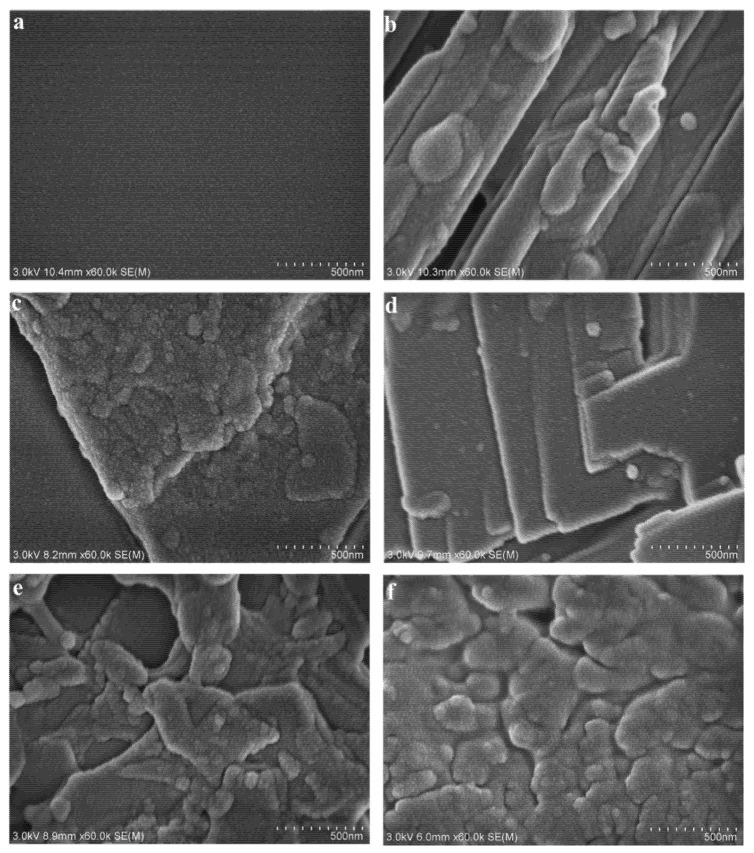
SEM images of (**a**) unmodified oil-wet calcite and of oil-wet calcite modified with (**b**) CTAB, (**c**) SDS, (**d**) TX-100, (**e**) rhamnolipid, and (**f**) sophorolipid.

SEM images of the original oil-wet calcite surface and the surfactant-treated oil-wet calcite surface are shown in Figure 12. CTAB, SDS, and TX-100 were the chemical surfactants used; their relative molecular mass was small; and the steric hindrance between different the surfactant molecules in aqueous solution was small. CTAB is a cationic surfactant; SDS is an anionic surfactant; and TX-100 is a non-ionic surfactant. CTAB created gullies in the same direction in the oil-wet calcite surface, and the overhang section was similar to a round ball. SDS made the oil-wet surface uneven. When treated with TX-100, the surface became bulky and showed regular leveling. The calcite surface showed an irregular and blocky shape. Due to the fact that the chemical surfactants showed a small relative molecular mass and small steric hindrance, they altered the oil-wet calcite surface mainly by adsorption. Furthermore, the oil-wet calcite surface became angular after chemical surfactant treatment; namely, a few heavy oil components were released from the surface by the chemical surfactants due to the angular shape. Additionally, the oil-wet calcite surface had a negative charge because the bitumen had a negative charge. As CTAB is a cationic surfactant, its adsorption was better. Meanwhile, rhamnolipid and sophorolipid are biosurfactants, and the relative molecular mass and steric hindrance between the biosurfactant molecules were high. Figure 12d,e show that the oil-wet calcite surface edges and corners became irregular, which means that some heavy oil components were freed from the mineral surface. We thus confirmed that rhamnolipid and sophorolipid could solubilize organic compounds [33,34,35]. In addition to the solubilization effect, some biosurfactant molecules could adsorb onto the oil-wet calcite surface. The surfactant molecules helped the heavy oil components to be released from the mineral surface and adsorbed onto the oil-wet calcite surface; the different surface topographies made the oil-wet surface show different wettabilities.

**Figure 13:**
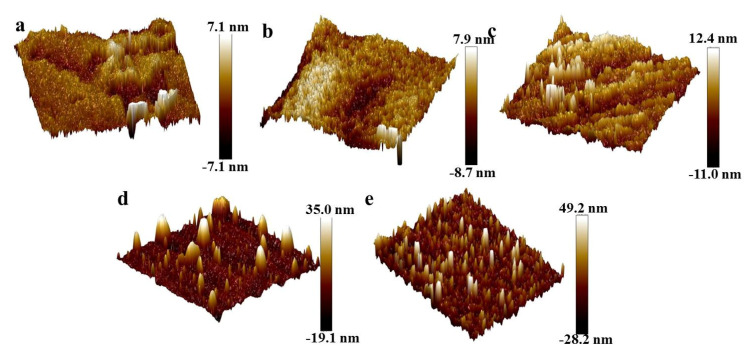
AFM images of oil-wet calcite modified with (**a**) CTAB, (**b**) SDS, (**c**) TX-100, (**d**) sophorolipid, and (**e**) rhamnolipid.

AFM images of the oil-wet calcite surface treated by five surfactants are shown in Figure 13. CTAB, SDS, TX-100, rhamnolipid, and sophorolipid treatments led to different oil-wet calcite surface topographies and roughness values. The oil-wet calcite surface became more hydrophilic when the surface roughness became higher in a certain range [19,31]. The oil-wet calcite surface roughness values after being treated by CTAB, SDS, and TX-100 were 7.1, 7.0, and 12.4 nm, respectively. However, the calcite surface roughness values following rhamnolipid and sophorolipid treatment were 49.2 and 35.0 nm, respectively. The roughness of oil-wet calcite treated by chemical surfactants was lower than that of the samples treated with biosurfactants, which was due to the fact that the biosurfactant molecules and steric hindrance were high. Figure 13a–c show that there were fewer humps on the oil-wet calcite treated by chemical surfactants as opposed to biosurfactants. The reason was that most chemical surfactants only adsorbed onto the oil-wet calcite surface, and the release of heavy oil was rare. Figure 13d,e show that the oil-wet calcite surface had many humps, which meant that many heavy oil components had been released from the oil-wet calcite surface, and the erosion effect was high, so the oil-wet calcite surface wettability altered significantly.

#### 3.5.2. Elemental Analysis

The results from the EDS analysis of figures of oil-wet calcite modified by five surfactants are shown in Table 3. When the oil-wet calcite surface was treated with CTAB, N and Br element peaks were shown on the oil-wet calcite surface, which indicated that CTAB could effectively adsorb on the oil-wet calcite surface. SDS, TX-100, sophorolipid, and rhamnolipid could adsorb onto the oil-wet calcite surface for the same reason. Except for sophorolipid, when the oil-wet calcite was treated by other surfactants, the amount of C on the calcite surface decreased. However, when the oil-wet calcite surface was treated with sophorolipid, the C content on the calcite surface increased, due to the high molecular weight of sophorolipid and the high C content.

### 3.6. Nanofluids’ Effect on Oil-Wet Calcite Wettability

#### 3.6.1. Nanofluid Species Effect

**Figure 14:**
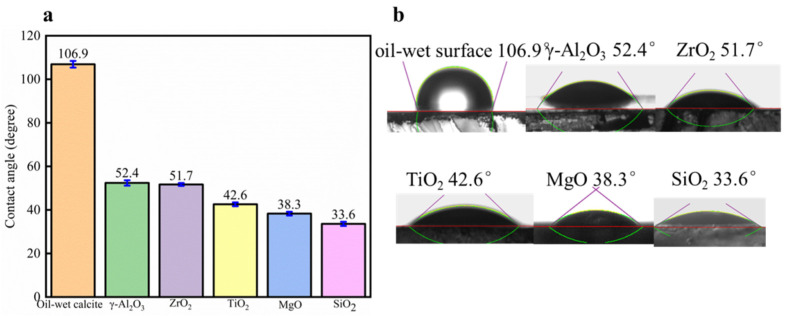
The effects of the different nanofluid species on oil-wet calcite wettability (25 °C). (**a**) Contact angle histogram; (**b**) Measurement figure.

As shown in Figure 14, when the oil-wet calcite surface was treated by five different nanofluids—namely γ-Al_2_O_3_, ZrO_2_, TiO_2_, MgO, and SiO_2_—the oil-wet calcite became more hydrophilic, and the hydrophilicity gradually increased. γ-Al_2_O_3_ treatment reduced the oil-wet calcite surface contact angle from 106.9° to 55.9°, and its effect was the lowest among the five nanofluids. SiO_2_ treatment made the oil-wet calcite wettability more hydrophilic, and the water contact angle was 33.6°, thus showing that the effect of SiO_2_ was optimal. The different nanofluids had different adsorption effects on the mineral surface, due to the fact that they have different surface morphologies and charge distributions [20]. Hence, at the same concentration, the different nanofluids had different effects on the oil-wet calcite surface.

#### 3.6.2. Nanofluid Concentration Effect

**Figure 15:**
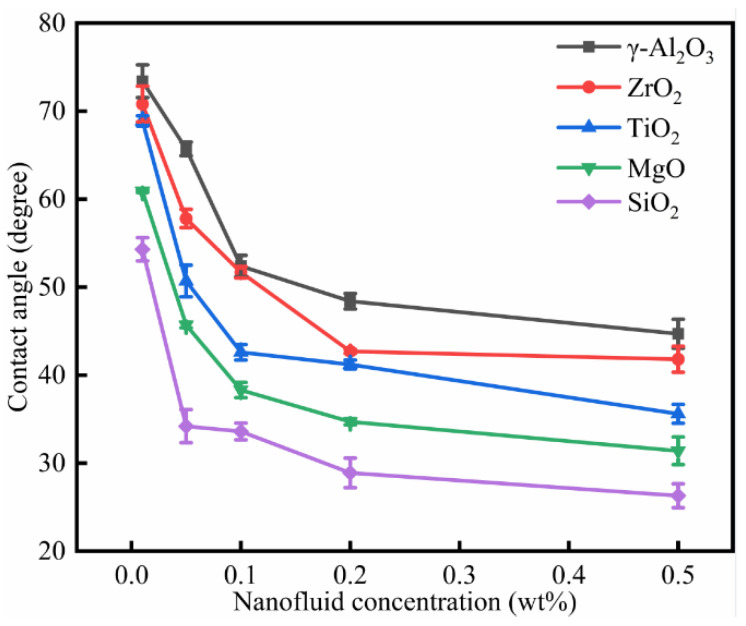
The nanofluid concentration effect on oil-wet calcite wettability (25 °C).

As shown in Figure 15, the oil-wet calcite surface became more hydrophilic when the nanofluid concentration was increased. When the concentration of nanofluid was increased, its adsorption on the mineral surface increased, and the calcite surface became gradually covered by nanoparticles. When the nanofluid concentration was 0.1 wt%, the nanofluid adsorption level reached saturation. When the nanofluid concentration reached 0.2 wt%, the contact angle gradually stabilized. In consideration of the nanofluids’ cost and the economic benefits, 0.1 wt% was determined as the optimal concentration. For the same given concentration, the decrease in water contact angle on the oil-wet calcite surface was according to the following order: γ-Al_2_O_3_ > ZrO_2_ > TiO_2_ > MgO > SiO_2_. As shown in Figure 11, the roughness measurements of the oil-wet calcite surfaces treated by SiO_2_, MgO, ZrO_2_, TiO_2_, and γ-Al_2_O_3_ nanofluids were 113.1, 45.1, 121.5, 21.7, and 37.33 nm, respectively. The roughness influenced the surface wettability, and higher roughness was beneficial to water-wet surfaces, but the influence was only one-sided. Figure 10 shows that the coverage of nanoparticles influenced the mineral surface wettability, and a higher coverage of nanoparticles was beneficial to water-wet surfaces. Besides surface roughness and nanoparticles coverage, the nanoparticle adsorption properties and surface energy influenced mineral wettability. SiO_2_ nanoparticles showed optimal adsorption [36] and thus had an optimal effect on the oil-wet calcite wettability.

#### 3.6.3. Temperature Effect

**Figure 16:**
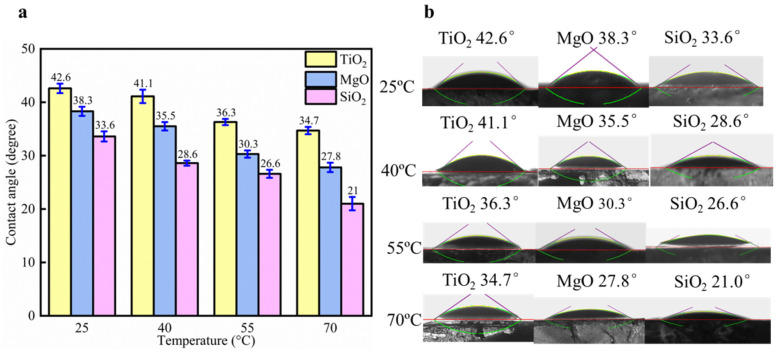
Temperature effect on oil-wet calcite for 0.1 wt% TiO_2_, MgO, and SiO_2_ nanofluids. (**a**) Contact angle histogram; (**b**) Measurement figure.

The effect of temperature on oil-wet calcite for 0.1 wt% TiO_2_, MgO, and SiO_2_ nanofluids is shown in Figure 16. The contact angle decreased when the temperature of the same nanofluids increased. When the temperature increased from 25 to 70 °C, the contact angle (by TiO_2_ nanofluids) decreased from 42.6° to 34.7°. The contact angle (by MgO nanofluids) decreased from 38.3° to 27.8° when the temperature increased from 25 to 70 °C. The contact angle (by SiO_2_ nanofluids) decreased from 33.6° to 21° when the temperature increased from 25 to 70 °C. At the same temperature, the contact angle on the oil-wet calcite surface treated with different nanofluids followed the order of TiO_2_ > MgO > SiO_2_. When the temperature increased, the adsorption ability of nanofluids along with their movement speed also increased. Their adsorption effect was also enhanced, which results in oil-wet calcite wettability alterations [37,38]. Furthermore, when the temperature increased, the heavy oil viscosity decreased, which enhanced the oil–solid separation process [39]. The literature indicates that nanoparticle adsorption is irreversible [19]. Due to the fact that nanoparticle desorption was ignored, the effect of temperature on the mineral surface wettability could be ignored.

#### 3.6.4. Salinity Effect

**Figure 17:**
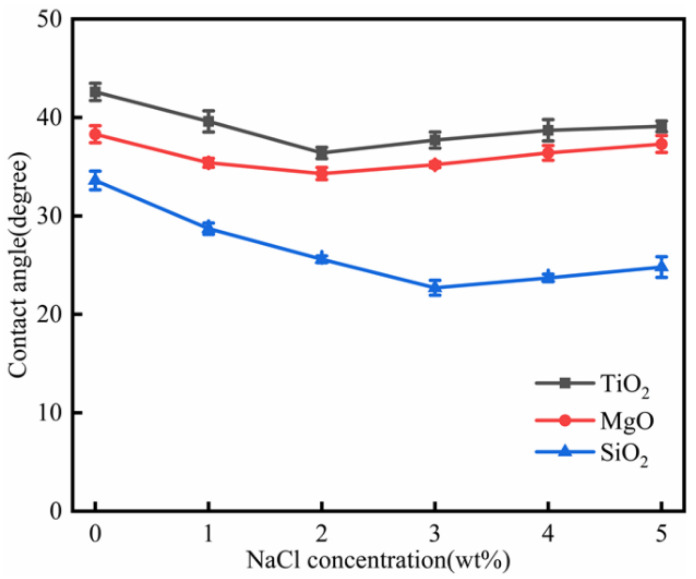
The effect of TiO_2_, MgO, and SiO_2_ nanofluids on oil-wet calcite wettability at different salinities.

The effect of TiO_2_, MgO, and SiO_2_ nanofluids on the oil-wet calcite wettability at different salinities is shown in Figure 17. When the nanofluid concentration did not reach the optimal concentration, the contact angle decreased with higher salinity. However, when the salinity increased further, the contact angle increased. For different nanofluids, the optimal salinity was different. The optimal salinity for SiO_2_, TiO_2_, and MgO nanofluids was 3, 2, and 2 wt%, respectively. The nanofluids remained stable at low salinity; however, the nanofluids became unstable when the salinity increased [40], because higher salinity results in compression of the electric double layers, which increased the adsorption rate of nanoparticles. Therefore, when the salinity was lower than the optimal level, the contact angle decreased due to nanofluid instability. However, when the salinity reached the optimal level, the contact angle increased slightly, due to adsorption of Na^+^ onto the oil-wet calcite surface [41], and its occupation of adsorption sites further increases nanoparticle adsorption. Therefore, the contact angle would increase further [42].

### 3.7. Surfactants’ Effect on Oil-Wet Calcite Wettability

**Figure 18:**
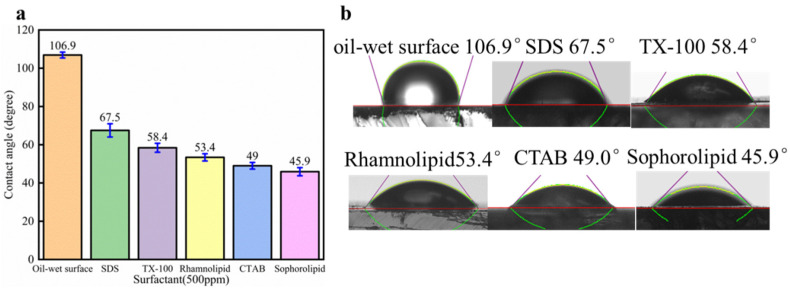
Effect of surfactants alone on oil-wet calcite wettability. (**a**) Contact angle histogram; (**b**) Measurement figure.

The effect of surfactants alone on oil-wet calcite wettability is shown in Figure 18. Five surfactants could make the oil-wet calcite surface more hydrophilic, and the water contact angle was as follows: sophorolipid (45.9°) > CTAB (49°) > rhamnolipid (53.4°) > TX-100 (58.4°) > SDS (67.5°). The reason was that different surfactants have different oil-solid separation abilities and different adsorption properties. The five surfactants’ heavy oil recovery was as follows: sophorolipid > CTAB > rhamnolipid > SDS > TX-100. Different surfactants showed different effects on the recovery of heavy oil, and the surfactants’ effects on carbonate rock wettability differed significantly. When the carbonate rocks became more hydrophilic, the heavy-oil–solid separation efficiency increased, resulting in increased heavy oil recovery.

### 3.8. Zeta Potential Measurement

**Figure 19:**
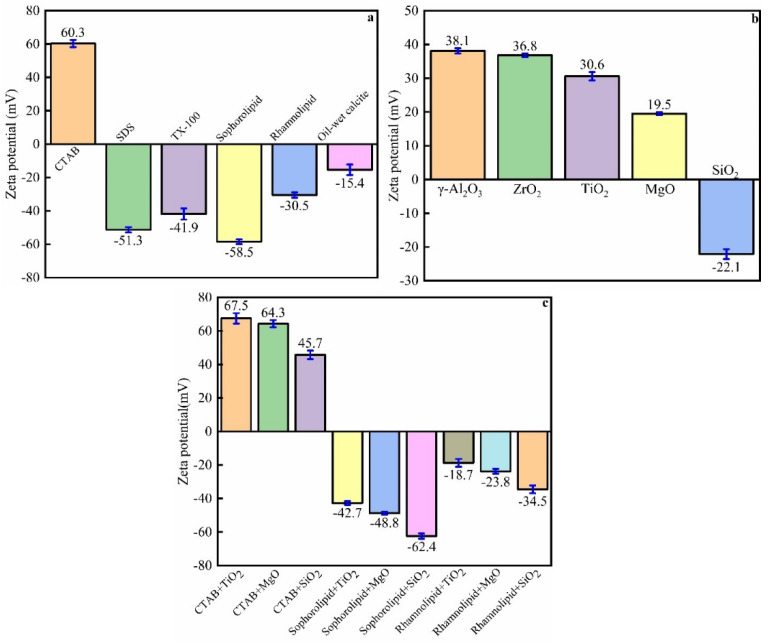
Zeta potential of (**a**) 500 ppm surfactants; (**b**) 0.1 wt% nanofluids; (**c**) 500 ppm surfactant and 0.1 wt% nanofluid hybrid solutions.

The zeta potential of the surfactants, nanofluids, and surfactant–nanofluid hybrid solutions is shown in Figure 19. The zeta potential of 500 ppm CTAB, SDS, TX-100, sophorolipid, and rhamnolipid was 60.3, −51.3, −41.9, −58.5, and −30.5 mV, respectively. The oil-wet calcite had a negative charge, and its zeta potential was −15.4 mV due to the fact that bitumen has a negative charge. The zeta potential of 0.1 wt% γ-Al_2_O_3_, ZrO_2_, TiO_2_, MgO, and SiO_2_ nanofluids was 38.1, 36.8, 30.6, 19.5, and −22.1 mV, respectively. When the nanofluids and surfactants were combined together, the zeta potential values were similar to those of the surfactants. The surfactants adsorbed onto the nanoparticle surface, so the zeta potential was nearly the same as that of the surfactants. If the zeta potential of the surfactants and nanofluids had opposing charges, the surfactant–nanofluid hybrid solution zeta potential would decrease.

### 3.9. Synergistic Effect of Nanofluids and Surfactants on Oil-Wet Calcite Wettability

**Figure 20:**
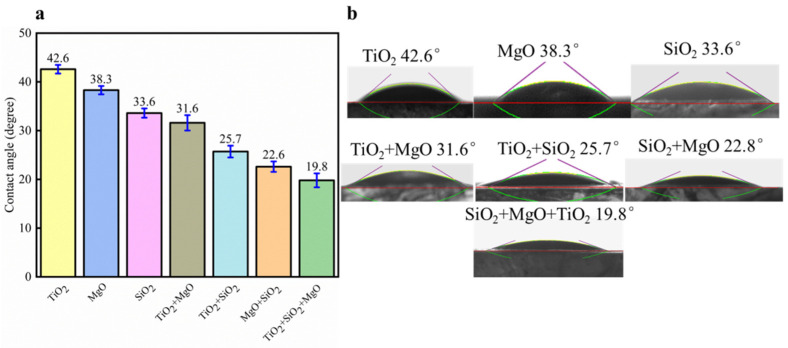
Effect of various nanofluid mixtures on oil-wet calcite wettability. (**a**) Contact angle histogram; (**b**) Measurement figure.

The nanoparticle formulations could effectively alter the oil-wet calcite wettability, as shown in Figure 20. On the oil-wet calcite surface treated with hybrid nanofluids, the water contact angle decreased, and the wettability became more hydrophilic. When two species of nanofluids were blended, the water contact angle became lower than with any one of the two nanofluids. When the oil-wet calcite surface was treated with TiO_2_ and MgO, the water contact angle became 31.6°, which was lower than the contact angle with TiO_2_ (42.6°) and MgO (38.3°). When TiO_2_ and SiO_2_ were blended, the contact angle on the oil-wet calcite was 25.7°, which was lower than the contact angle with TiO_2_ (42.6°) and SiO_2_ (33.6°). When SiO_2_ and MgO were blended, the contact angle on the oil-wet calcite was 22.8°, which was lower than the contact angle with SiO_2_ (33.6°) and MgO (38.3°). When SiO_2_, MgO, and TiO_2_ nanoparticles were combined together, the contact angle of the oil-wet calcite surface decreased to 19.8°. Thus, hybrid nanofluids could effectively alter the oil-wet calcite surface wettability, due to the following reasons [41,43]: Firstly, different species of nanoparticles have different shapes and sizes. Compared to the single nanoparticles, the shape of the hybrid nanofluids became more compact and showed a uniform distribution when different nanoparticles were combined. The hybrid nanoparticles had a higher ability to adsorb onto the oil-wet calcite surface, especially onto small rough areas. Secondly, compared to the single nanofluids, the hybrid nanofluids became unstable, and the instability accelerated nanoparticle adsorption onto the oil-wet calcite surface. Thirdly, the hybrid nanoparticles had increased competitive adsorption, and the adsorption area increased due to electrostatic interactions. When one nanoparticle adsorbed onto the oil-wet calcite surface, there was an attractive force between other nanoparticles, which accelerated the adsorption of other nanoparticles onto the oil-wet calcite surface. In the end, the SiO_2_ and MgO hybrid nanofluids rendered the oil-wet calcite surface more hydrophilic, which means that the SiO_2_ and MgO combined nanofluid showed the optimal effect in enhancing adsorption.

**Figure 21:**
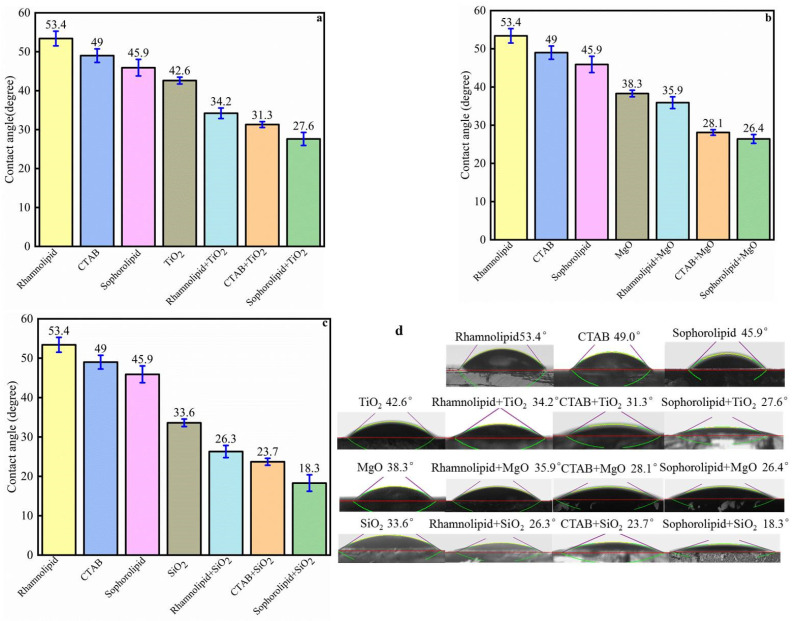
Synergistic effect of nanofluids and surfactants on oil-wet calcite wettability: (**a**) TiO_2_ and surfactants; (**b**) MgO and surfactants; (**c**) SiO_2_ and surfactants; (**d**) measurement figure.

The synergistic effect of nanofluids and surfactants on the oil-wet calcite surface wettability is shown in Figure 21. Following treatment with rhamnolipid, CTAB, or sophorolipid alone, the water contact angle on the oil-wet calcite was 53.4°, 49°, and 45.9°, respectively. Following treatment with TiO_2_, MgO, or SiO_2_ alone, the water contact angle on the oil-wet surface was 42.6°, 38.3°, and 33.6°, respectively. When nanoparticles were dispersed into the surfactants, surfactant–nanofluid complex solutions were created, and the contact angle decreased further. When TiO_2_ was dispersed into the rhamnolipid, CTAB, and sophorolipid surfactants, and the surfactant–nanofluid complex solutions formed, the water contact angle on the oil-wet calcite surface was 34.2°, 31.3°, and 27.6°, respectively, and was much lower than the water contact angle (42.6°) on the oil-wet calcite surface treated with TiO_2_ nanofluids alone. Additionally, the contact angle was much lower than that on the oil-wet calcite surface treated with either rhamnolipid, CTAB, or sophorolipid alone (53.4°, 49°, and 45.9°, respectively). SiO_2_ and MgO nanofluids had similar effects in that the surfactant–nanofluid complex solutions could increase the oil-wet calcite surface hydrophilicity; in other words, nanofluids and surfactants have a synergistic effect on heavy-oil–solid separation. The effect of the hybrid solution formed with SiO_2_ nanofluid and the biosurfactant sophorolipid was optimal, and the water contact angle on the oil-wet calcite surface was 18.3°.

### 3.10. Synergistic Effect Mechanism

**Figure 22:**
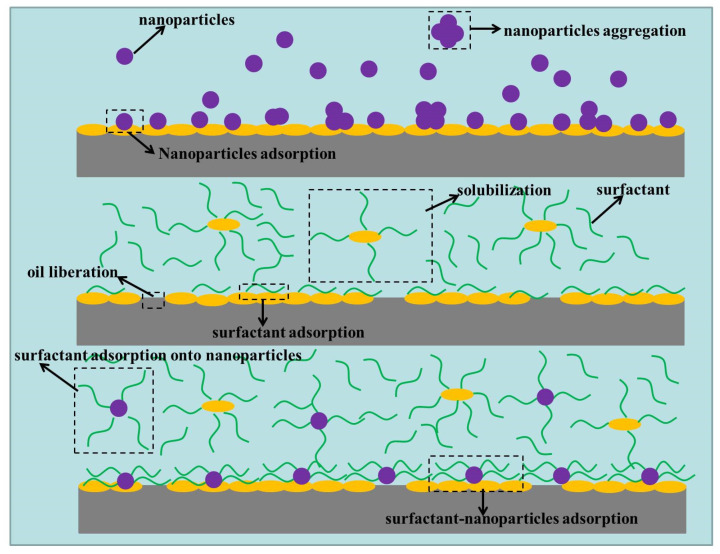
The mechanism of the synergistic effect of surfactants and nanofluids on oil-wet calcite surface wettability alteration.

The surfactant and nanofluid hybrid solutions could synergistically alter the oil-wet calcite wettability and make the oil-wet calcite surface become hydrophilic. Both nanofluids and surfactants alone could make the oil-wet calcite surface hydrophilic, but the mechanisms were different. When nanofluids alone were used to treat an oil-wet calcite surface, the nanoparticles would adsorb onto the surface [41,44]. The detailed mechanism is shown in Figure 22. The adsorption of nanoparticles was uneven due to their aggregation. The oil-wet calcite surface element composition and morphology were altered, which made the oil-wet calcite surface more hydrophilic. When surfactants alone were used to treat an oil-wet calcite surface, the role of surfactants could be divided into two parts. On the one hand, surfactants would adsorb onto the oil-wet calcite surface as with nanofluids [45,46,47]. On the other hand, surfactants could solubilize heavy oil molecules—that is, some heavy oil components would be released from the mineral surface—which made the water-wet calcite surface exposed [48,49].

When nanoparticles were dispersed into a surfactant solution, the surfactant molecules would adsorb onto the nanoparticle surface [44]. The bulk concentration of other surfactants decreased, and the steric hindrance between the surfactant molecules (bulk solution) decreased, which accelerated the surfactant molecules’ movement speed. Due to the fact that surfactants adsorbed onto the nanoparticle surface, the steric hindrance between nanoparticles increased, and the repulsive force between different nanoparticles increased; therefore, their movement speed increased. When nanoparticles were dispersed into deionized water, the nanoparticles aggregated, and the nanoparticle adsorption was uneven. However, the nanoparticles would not aggregate in surfactant solution, and the surfactant–nanoparticles adsorption became uniform, which made the oil-wet calcite surface more hydrophilic.

## 4. Conclusions

In this paper, we studied the synergistic effect of nanofluids and surfactants on heavy oil recovery and oil-wet calcite surface wettability alteration. Additionally, the synergistic effect on mineral wettability alteration was analyzed, and the detailed conclusions are as follows:

1. Nanofluids alone or surfactants alone could effectively alter oil-wet calcite surface wettability and render the oil-wet calcite surface more hydrophilic. The reasons were as follows: Nanoparticles could adsorb onto the mineral surface, which made the oil-wet calcite surface more hydrophilic. Furthermore, surfactant molecules could assist heavy oil to be released from the calcite surface, which exposed the surface. The surfactants could then adsorb onto the oil-wet calcite surface, and the nanofluids and surfactants could synergistically alter the oil-wet calcite surface wettability.
2. Surfactants alone could alter the oil-wet calcite surface wettability, and their effect in increasing the water contact angle occurred according to the following order: sophorolipid (45.9°) < CTAB (49°) < rhamnolipid (53.4°) < TX-100 (58.4°) < SDS (67.5°). Of the tested surfactants, sophorolipid showed the optimal effect on the oil-wet calcite surface.
3. Nanofluids alone could also alter oil-wet calcite surface wettability, and their effect in increasing oil-wet calcite wettability occurred according to the following order: SiO_2_ > MgO > TiO_2_ > ZrO_2_ > γ-Al_2_O_3_. Nanofluid concentration, salinity, and temperature influence the oil-wet calcite wettability; the optimal nanofluid concentration was 0.2 wt%, and the optimal salinity was 3 wt% for SiO_2_ nanofluids.

## Figures and Tables

**Table 1:** SEM–EDS analysis of original water-wet calcite and modified oil-wet calcite.

	Element	Weight Content (wt%)
Water-wet calcite	C_K_	6.46
	O_K_	50.23
	Ca_K_	43.31
Oil-wet calcite	C_K_	30.22
	O_K_	27.49
	Ca_K_	42.29

**Table 2:** EDS analysis of figures of oil-wet calcite modified using five nanofluids.

Treatment Method	Element	Weight Content (wt%)	Treatment Method	Element	Weight Content (wt%)
Oil-wet calcite	C	30.22	Al_2_O_3_ treat	C	7.56
	O	27.49		O	50.34
	Ca	42.29		Al	8.85
				Ca	33.25
ZrO_2_ treat	C	5.00	TiO_2_ treat	C	10.62
	O	47.05		O	47.87
	Zr	6.01		Ca	39.61
	Ca	41.93		Ti	1.90
MgO treat	C	7.48	SiO_2_ treat	C	4.85
	O	51.91		O	49.82
	Mg	1.55		Si	0.21
	Ca	39.06		Ca	45.12

**Table 3:** EDS analysis of figures of oil-wet calcite modified with five surfactants.

Treatment Method	Element	Weight Content (wt%)	Treatment Method	Element	Weight Content (wt%)
Oil-wet calcite	C	30.22	TX-100	C	17.37
	O	27.49		O	38.42
	Ca	42.29		Ca	44.21
CTAB	C	7.08	SDS treat	C	24.74
	N	1.66		O	32.09
	O	49.84		Na	0.72
	Br	0.06		S	1.78
	Ca	41.36		Ca	40.67
Sophorolipid	C	33.62	Rhamnolipid	C	17.68
	O	35.97		O	39.20
	Ca	30.41		Ca	43.12

## Data Availability

Data is contained within the article.
